# Pan-cancer analyses identify DCBLD2 as an oncogenic, immunological, and prognostic biomarker

**DOI:** 10.3389/fphar.2022.950831

**Published:** 2022-08-11

**Authors:** Pan Xie, Jun-Yan Liu, Han Yan, Zhi-Bin Wang, Shi-Long Jiang, Xi Li, Zhao-Qian Liu

**Affiliations:** ^1^ Department of Clinical Pharmacology, Hunan Key Laboratory of Pharmacogenetics, and National Clinical Research Center for Geriatric Disorders, Xiangya Hospital, Central South University, Changsha, China; ^2^ Institute of Clinical Pharmacology, Central South University, Changsha, China; ^3^ Department of Orthopaedics, Xiangya Hospital, Central South University, Changsha, China; ^4^ Department of Pharmacy, The Second Xiangya Hospital, Central South University, Changsha, China

**Keywords:** chemotherapy, DCBLD2, immunotherapy, prognosis, pan-cancer

## Abstract

Discoidin, CUB, and LCCL domain-containing protein 2 (*DCBLD2*) is a two-domain transmembrane protein-coding gene located on chromosome 3, the protein expressed by which acts as the membrane receptor of semaphorin and vascular endothelial growth factor during the development of axons and blood vessels. Although several research evidences at the cellular and clinical levels have associated DCBLD2 with tumorigenesis, nothing is known regarding this gene from a pan-cancer standpoint. In this study, we systematically analyzed the influence of DCBLD2 on prognosis, cancer staging, immune characteristics, and drug sensitivity in a variety of cancers based on a unified and standardized pan-cancer dataset. In addition, we performed GO enrichment analyses and KEGG analyses of *DCBLD2*-related genes and DCBLD2-binding proteins. Our results showed that DCBLD2 is a potential oncogenic, immunological as well as a prognostic biomarker in terms of pan-cancer, and is expected to contribute to the improvement of tumor prognosis and the development of targeted therapy.

## Background

The pan-cancer analysis contributes to examining similarities and differences between genotypic and phenotypic characteristics across different kinds of cancer. Since tumorigenesis and progression are affected by many factors, there is a need to study and elaborate on the pan-cancer influence of candidate genes and discuss the correlation between gene expression and patient outcome, tumor malignancy, immune regulation, and drug sensitivity as well as the potential underlying molecular mechanisms. Datasets held at the TCGA database contain invaluable functional genomic information for different tumors, which lays a foundation for gene pan-cancer analysis (Weinstein et al., 2013; Blum et al., 2018; Campbell et al., 2020).

Discoidin, CUB and LCCL Domain Containing 2 (*DCBLD2*) was first cloned from lung cancer cells with strong metastatic ability by Koshikawa et al. (Koshikawa et al., 2002) and found to be significantly up-regulated in high, relative to low, metastatic lung cancer cell lines. The DCBLD2 protein contains a binding peptide that is partially homologous to the domain of the SEMA4B signal element. Previous co-immunoprecipitation results confirmed that DCBLD2 interacts with SEMA4B-Fc and full-length SEMA4B, evidence that laid a foundation for subsequent explorations into the function and underlying mechanism of DCBLD2 action (Nagai et al., 2007). Furthermore, it was demonstrated that the over-expression of DCBLD2 is related to the increased invasive capacity of cancer cells and poor prognosis of patients with lung cancer (Koshikawa et al., 2002; Nagai et al., 2007), gastric cancer (Kim et al., 2008), colorectal cancer (He et al., 2020), and pancreatic cancer (Raman et al., 2018; Feng et al., 2020).

The tumor microenvironment (TME) is composed of cancer cells, stromal cells, immune cells, secretory products (such as cytokines and chemokines) of corresponding cells, and extracellular matrix (ECM). Cancer cells and their survival microenvironment are interdependent but antagonistic. TME can affect and regulate the occurrence and development of tumor through changes in metabolism, secretion, immunity, structure and function. As a consequence, researching the function of TME plays an important role in tumor diagnosis, prevention and treatment (Whiteside, 2008; Wu and DAI, 2017). Notably, the TME contains a variety of cells, of which infiltrating immune cells, such as T cells, B cells, macrophages, natural killer cells, and dendritic cells, account for the largest proportion. Different tumors can develop immune tolerance by escaping the effective recognition and killing of cancer cells by the immune regulation, while immunotherapy restores the normal anti-tumor immune response by restarting immune regulation in the tumor microenvironment, to inhibit tumor growth and metastasis. This treatment has shown strong antitumor ability in a variety of cancers such as melanoma, lung cancer, kidney cancer, and prostate cancer (Gajewski et al., 2013; Topalian et al., 2015). Apart from immune cells, infiltration of other kinds of cells such as cancer-associated fibroblasts (CAFs) and vascular endothelial cells inside the tumor has also been documented, a phenomenon that constitutes a non-immune microenvironment of the tumor. For example, CAFs have been proved to release stromal cell-derived factors and proangiogenic growth factors which in turn promote the progression and metastasis of cancer (Monteran and EREZ, 2019).

At present, nothing is known regarding systematic regulation of DCBLD2 on tumorigenesis, prognosis, and drug susceptibility in pan-cancer, while knowledge on the relationship between DCBLD2 and TME, including immune infiltration, is still lacking. Here, we provide the first report of the effects of DCBLD2 on tumorigenesis, prognosis, immune infiltration, and drug sensitivity in pan-cancer based on a dataset from TCGA. Our findings indicate that DCBLD2 is an oncogenic, immunological, and prognostic factor and has the potential to be a biomarker for cancer diagnosis, drug development, and prognostic analyses.

## Materials and methods

### Online analysis tools used in this study

The databases used in this study and their web addresses are listed in the table below (Table 1).

### Differential gene expression analysis

We downloaded a unified and standardized pan-cancer data set, namely the TCGA Pan-Cancer (PANCAN, N=10535, G=60499) from UCSC ( https://xenabrowser.net/), and extracted expression data for the *DCBLD2* gene. Next, we screened the samples from Solid Tissue Normal, Primary Blood-Derived Cancer-Peripheral Blood, and Primary Tumor, and carried out log2 (x + 1) transformation on each expression value. All cancer species with less than 3 samples per single cancer species were excluded from the analysis to finally obtain an expression matrix comprising 26 cancer species. Differential expression between normal and tumor samples for each tumor was calculated using packages implemented in R software (version 3.6.4), with differences between groups determined using unpaired Student’s *t*-Test.

### Prognostic value analysis of DCBLD2

The TCGA dataset was downloaded as described above, while metastatic samples were screened from TCGA-LAML, Primary Tumor, and TCGA-SKCM. Next, we screened data from a previous study (Liu et al., 2018b) and retrieved high-quality TCGA prognostic datasets and samples with a follow-up period of fewer than 30 days. Each expression value was transformed by log2 (x + 1) transformation, and any cancer species with less than 10 samples were excluded from the analysis. Finally, we obtained expression data for 39 cancer species and the overall survival or disease-specific survival data of the corresponding samples were obtained. The Coxph function implemented in the survival package of R software (version 3.2-7) was utilized to establish a Cox proportional hazards regression model for the determination of the association between gene expression and prognosis in each tumor. Prognostic significance was obtained by Log-rank test statistical test.

### Immunophenoscore analysis

Data retrieval and screening of metastatic samples were as described above. Gene expression profiles in each tumor were extracted and mapped to Gene Symbol using the IOBR package (version 0.99.9) in R software (Method: Deconvo-IPS) (Charoentong et al., 2017). Next, MHC, EC, SC, CP, AZ, and IPS infiltration scores for each patient in each tumor were reevaluated according to the patterns of gene expression. This revealed a total of 6 types of immune cell infiltration scores across 9,555 and 39 tumor samples and tumor types, respectively. Next, we used the corr. test function in psych package (version2.1.6) in R software to calculate the Spearman’s correlation coefficient of gene and immune cell infiltration scores in each tumor and determine significantly related immune infiltration scores.

### Microenvironment cell populations-counter analysis

Data retrieval and initial manipulation were as earlier described above. Next, we employed the IOBR package (version 0.99.9) in R (Method: Deconvo-MCPcounter) (Becht et al., 2016). to evaluate infiltration of T cells, CD8 T cells, Cytotoxic lymphocytes, B lineage, NK cells, Monocytic lineage, Myeloid dendritic cells, Neutrophils, Endothelial cells and Fibroblasts for each patient across each tumor based on gene expression profiles. This revealed a total of 10 kinds of immune cell infiltration scores, across 9,555 tumor samples from 39 tumor types. Thereafter, we employed the corr. test function implemented in psych package (version 2.1.6) in R to calculate the Spearman’s correlation coefficient between *DCBLD2* expression level and immune cell infiltration scores in each tumor. Finally, we determined immune infiltration scores significantly related to *DCBLD2* expression.

### Estimation of stromal and immune cells in malignant tumor tissues using expression data analysis

Data retrieval and initial manipulation were as earlier described above. Next, we employed the ESTIMATE package in R (Yoshihara et al., 2013) to calculate stromal, immune, and ESTIMATE scores of each patient in each tumor and obtained immune infiltration scores for 9,555 and 39 tumor samples and tumor types, respectively. Next, we used the corr. test function implemented in psych package (version 2.1.6) in R to calculate the Pearson’s correlation coefficient between *DCBLD2* expression level and immune invasion scores in each tumor, then determined the significantly related immune invasion score.

### DCBLD2-related gene enrichment analysis

First of all, we used the STRING database to find the target proteins that may bind to DCBLD2. We set specific parameters on the “Basic Settings” page: “evidence” in the “Meaning of network edges” option, “Experiments” in the “Active interaction sources” option, and “low confidence (0.150)” in the “Minimum required interaction score” option, and “no more than 50 interactors” in the “Max number of interactors” option. When the condition is set, click “update” to get the protein interaction network. Next, we used GEPIA 2.0 to screen out the first 100 genes related to *DCBLD2* expression, and use the “correlation analysis” module to analyze the correlation between the top 10 genes and *DCBLD2* expression. The genes obtained from the above two databases were collected and merged, and the DAVID database was used for GO enrichment analysis and KEGG pathway analysis.

## Results

### Profiles of DCBLD2 expression, cellular localization, topology, and its correlation with diseases

**FIGURE 1:**
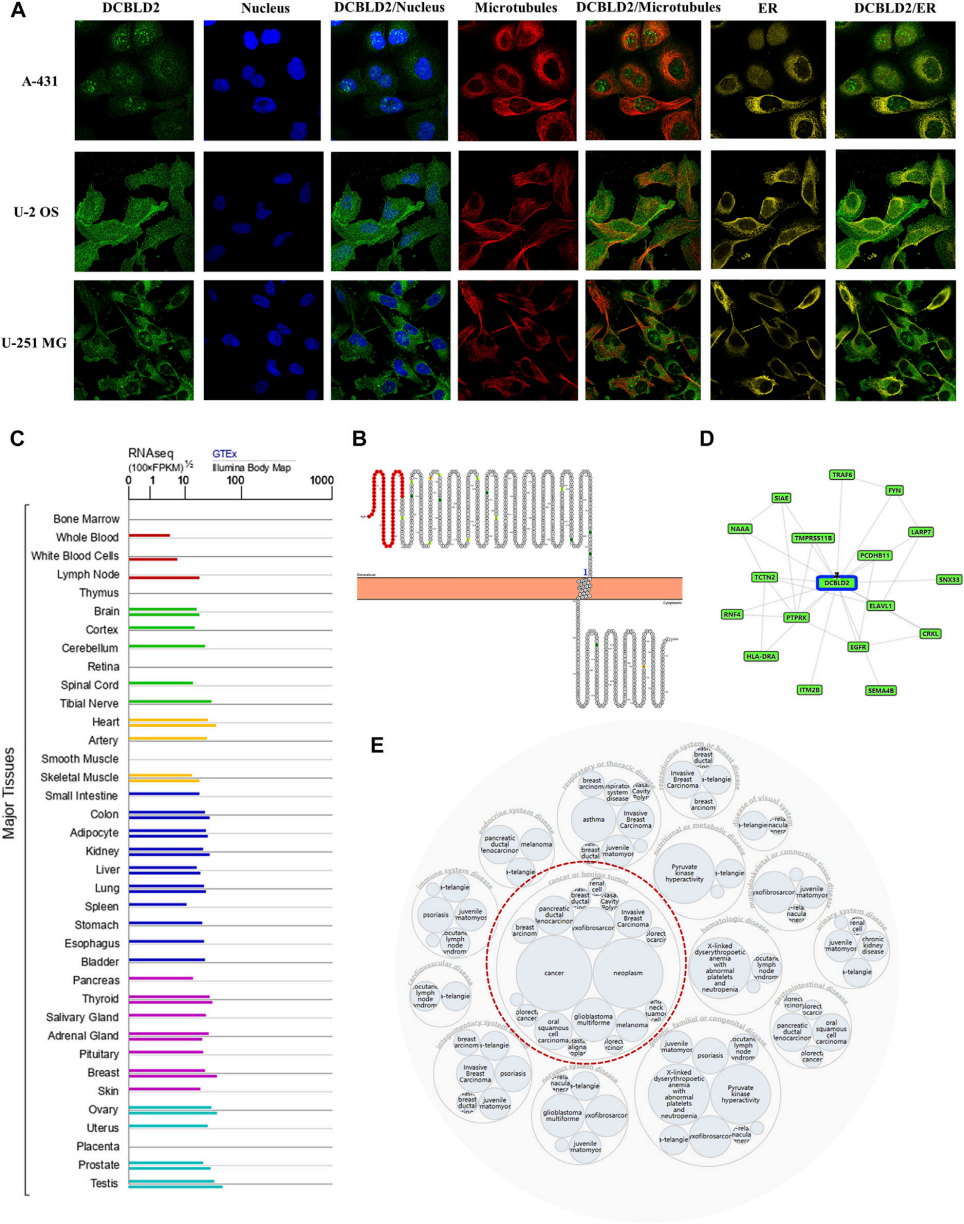
Cellular localization, topology, tissue expression of *DCBLD2* and its correlation with diseases. **(A)** Immunofluorescence assay showed intracellular localization of DCBLD2 protein in A-431, U-2 OS, and U-251 MG cells as adopted from the HPA database. **(B)** DCBLD2 protein topology showing transmembrane localization. **(C)** The mRNA expression level of *DCBLD2* in all normal human tissues as adopted from the TCGA data. **(D–E)** Network of potential binding proteins of DCBLD2 **(D)** and the *DCBLD2*-related disease **(E)**.

To characterize DCBLD2’s intracellular localization, we evaluated its distribution in the endoplasmic reticulum and microtubules of A-431, U-2 OS, and U-251 MG cells using data from the Human Protein Atlas database. Immunofluorescence assay results revealed an overlap between DCBLD2 and ER and microtubule across these cell types. The DCBLD2 protein was mainly expressed in the plasma membrane, and partly in the Golgi apparatus and the cytosol (Figure 1A). Results from analysis of the topological structure showed that the protein is a transmembrane protein, with a mutation site both inside and outside the membrane (Figure 1B). Furthermore, expression profiles showed that *DCBLD2* mRNA was expressed in normal human tissues and organs except the bone marrow (Figure 1C). Protein-protein interaction networks showed that DCBLD2 interacted with multiple targets, including EGFR, FYN, ELAVL1, and HLA-DRA (Figure 1D). Moreover, the disease interaction network revealed that this gene was associated with many diseases, including cancer, genetic diseases, nervous system disease, and metabolic disease among others, of which the association with cancer was the most significant (Figure 1E).

### DCBLD2 is not only aberrantly overexpressed but is associated with tumor stages

**FIGURE 2:**
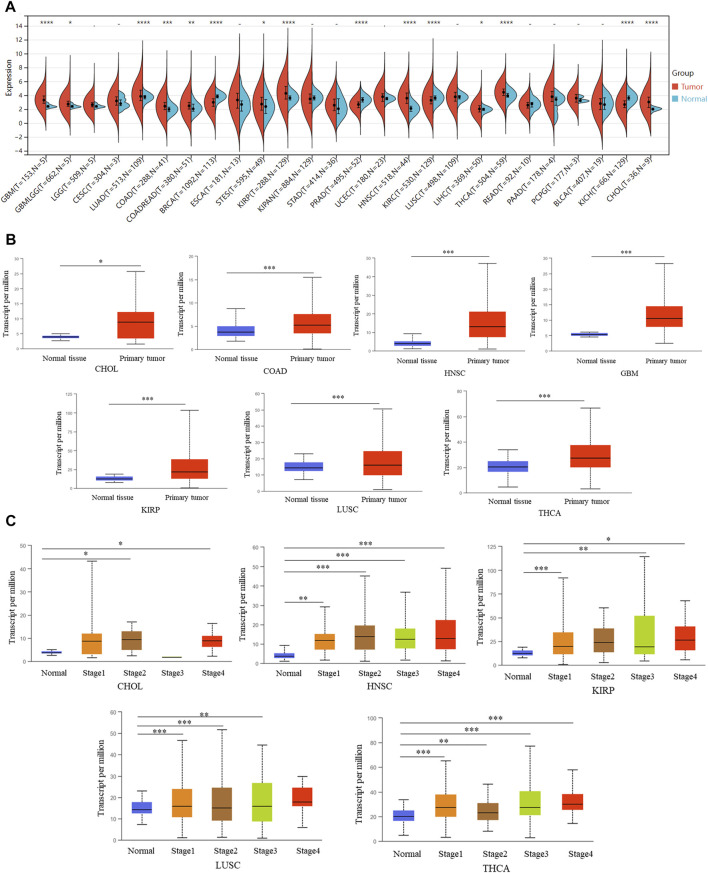
The association of the aberrant overexpression of *DCBLD2* with tumor stages. **(A)** Differential *DCBLD2* expression levels (log2 x + 1) between tumor and adjacent normal tissues as adopted from TCGA data. **(B)** Differential *DCBLD2* expression for the type of CHOL, COAD, GBM, HNSC, KIRP, LUSC, and THCA as adopted from TCGA data. *p < 0.05, **p < 0.01, **p < 0.001. **(C)** For the type of CHOL, HNSC, KIRP, LUSC and THCA in the TCGA project, the box plot data of differential *DCBLD2* expression levels (log2 x + 1) between pathological stages (stages I, II, III, and IV) were supplied. *p < 0.05, **p < 0.01, **p < 0.001.

*DCBLD2* was significantly over-expressed in most tumors, relative to normal adjacent tissues, including GBM (*p* = 1.8e-5), GBMLGG (*p* = 0.01), LUAD (*p* = 1.2e-8), COAD (*p* = 2.3e-4), COADREAD (*p* = 3.6e-3), STES (*p* = 0.03), KIRP (*p* = 1.4e-16), HNSC (*p* = 3.2e-20), LIHC (*p* = 0.02), THCA (*p* = 2.5e-8) and CHOL (*p* = 5.0e-5). Conversely, this gene was significantly downregulated in four kinds of tumors, relative to normal adjacent tissues, namely BRCA (*p* = 2.2e-40), PRAD (*p* = 9.1e-14), KIRC (*p* = 6.4e-6), and KICH (*p* = 4.6e-10) (Figure 2A). Validation of these results using the UALCAN database showed that the *DCBLD2* mRNA was significantly up-regulated in CHOL, COAD, GBM, HNSC, KIRP, LUSC, and THCA (*p* < 0.05) (Figure 2B). Results from differential expression of *DCBLD2* across different tumor stages showed that this gene was significantly upregulated in highly malignant pathological tumors including CHOL, HNSC, KIRP, LUSC, and THCA (*p* < 0.05) (Figure 2C). The above results showed that the expression levels of *DCBLD2* gene showed significant differences between tumor tissues and normal tissues in most cancers, suggesting that this gene has the potential to become a biomarker for evaluating the development of malignant tumors.

### DCBLD2 expression is associated with poor prognosis of cancer patients

**FIGURE 3:**
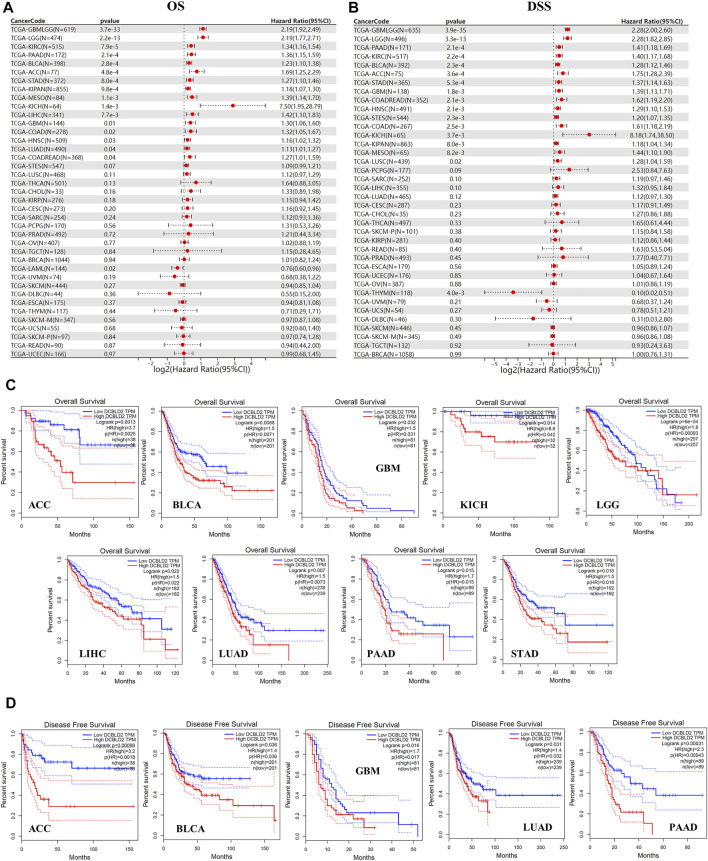
Association of *DCBLD2* with tumor-poor cancer prognoses. **(A)** Forest plot showing the correlation between *DCBLD2* expression and Overall Survival (OS) in 39 types of tumors as adopted from TCGA data. **(B)** Forest plot showing the correlation between *DCBLD2* expression and Disease-Specific Survival (DSS) in 39 types of tumors as adopted from TCGA data. **(C–D)** Analysis of the Overall Survival **(C)** and Disease-Free Survival **(D)** of multiple tumors based on *DCBLD2* expression as adopted from GEPIA 2.0 database (All *p* < 0.05).

We analyzed the influence of DCBLD2 expression on overall survival (OS) of patients with different tumor types via TCGA dataset. Results showed that upregulation of this gene was related to shorter OS of patients across 16 tumor types, namely GBMLGG (*p* = 3.7e-33, HR = 2.19), LGG (*p* = 2.2e-13, HR = 2.19), LUAD (*p* = 0.04, HR = 1.13), KIPAN (*p* = 9.8e-4, HR = 1.18), STAD (*p* = 8.0e-4, HR = 1.27), HNSC (*p* = 0.03, HR = 1.16), GBM (*p* = 0.01, HR = 1.30), KIRC (*p* = 7.9e-5, HR = 1.34), COAD (*p* = 0.02, HR = 1.32), COADREAD (*p* = 0.04, HR = 1.27), LIHC (*p* = 7.7e-3, HR = 1.42), MESO (*p* = 1.1e-3, HR = 1.39), PAAD (*p* = 2.1e-4, HR = 1.36), BLCA (*p* = 2.8e-4, HR = 1.23), ACC (*p* = 4.8e-4, HR = 1.69) and KICH (*p* = 1.4e-3, HR = 7.50). The only exception was recorded in LAML (*p* = 0.02, HR = 0.76) where high gene expression was significantly associated with increased OS (Figure 3A).

Similarly, analysis of the relevance between *DCBLD2* expression with disease specific survival (DSS) of patients revealed that high gene expression was significantly related to shorter DSS of patients in 16 tumor types, including GBMLGG (*p* = 3.9e-35, HR = 2.28), LGG (*p* = 3.3e-13, HR = 2.28), COAD (*p* = 2.5e-3, HR = 1.61), COADREAD (*p* = 2.1e-3, HR = 1.62), STES (*p* = 2.3e-3, HR = 1.20), KIPAN (*p* = 8.0e-3, HR = 1.18), STAD (*p* = 5.3e-4, HR = 1.37), HNSC (*p* = 2.1e-3, HR = 1.29), GBM (*p* = 1.8e-3, HR = 1.39), KIRC (*p* = 2.2e-4, HR = 1.40), LUSC (*p* = 0.02, HR = 1.28), MESO (*p* = 8.2e-3, HR = 1.44), PAAD (*p* = 2.1e-4, HR = 1.41), BLCA (*p* = 2.3e-4, HR = 1.28), ACC (*p* = 3.6e-4, HR = 1.75) and KICH (*p* = 3.7e-3, HR = 8.18). The only exception was in THYM (*p* = 4.0e-3, HR = 0.10) where high gene expression was associated with higher DSS (Figure 3B). Validation of the above results using the UALCAN database revealed that upregulation of *DCBLD2* was associated with significantly shorter OS of patients with ACC, BLCA, GBM, KICH, LGG, LIHC, LUAD, PAAD and STAD (*p* < 0.05) (Figure 3C), and significantly shorter DFS in patients with ACC, BLCA, GBM, LUAD and PAAD (*p* < 0.05) (Figure 3D). Furthermore, validation of these results using multiple GSE datasets revealed that high *DCBLD2*expression was significantly associated with shorter OS, DFS, DSS and RFS in multiple cancers, including colorectal, lung, breast, bladder and ovarian cancers (Supplymentary Figure S1). Based on these results, it is evident that the *DCBLD2* gene is a potential biomarker for patient outcomes.

### DCBLD2 is associated with activation of the EMT signal

**FIGURE 4:**
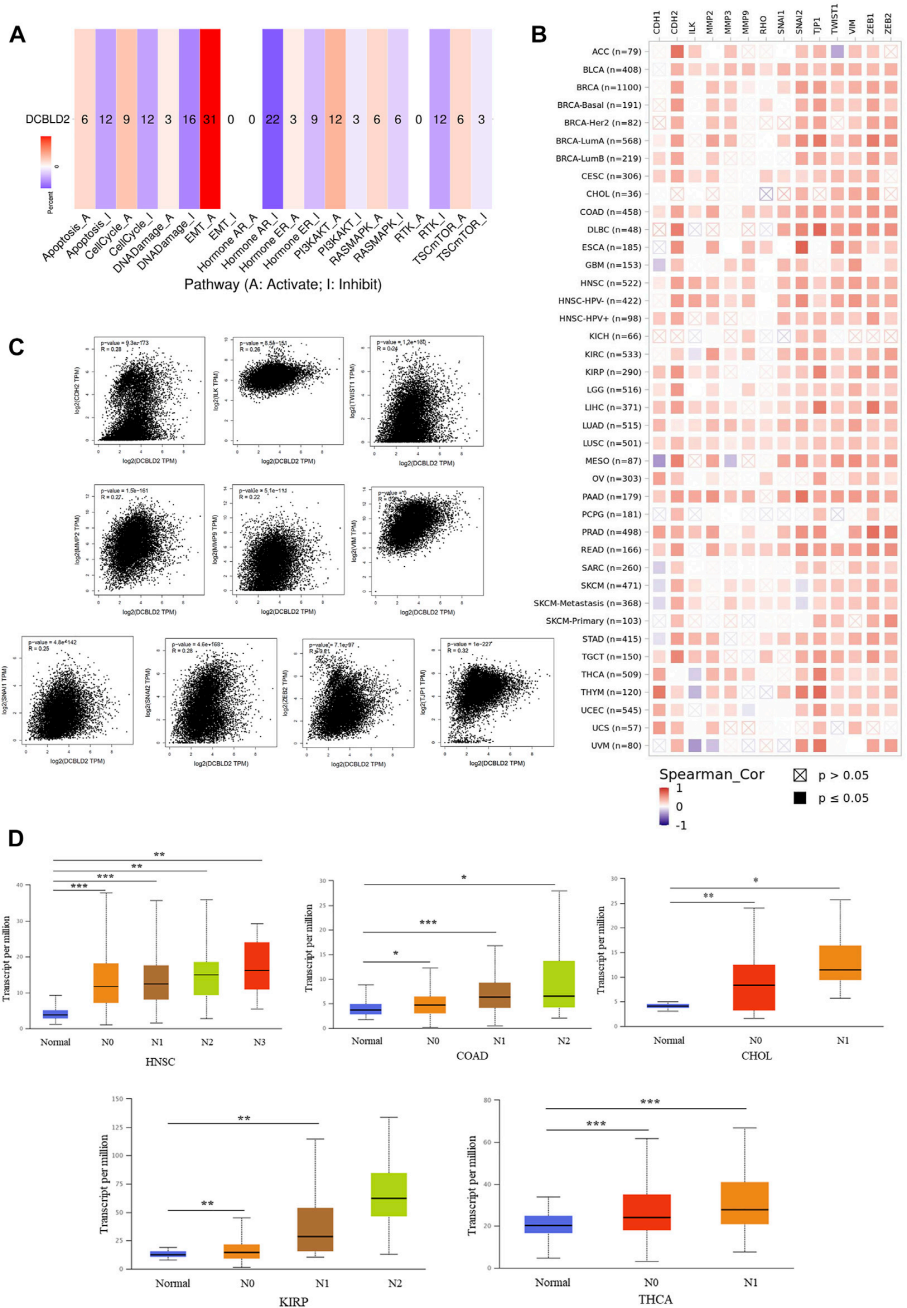
The association of *DCBLD2* with EMT signal activation. **(A)** The percentage of cancers in which mRNA expression of *DCBLD2* has a potential effect on pathway activity (A: Activate; I Inhibit). **(B)** The correlation heatmap of *DCBLD2* and the key regulators of EMT signal in the detailed cancer types. **(C)** Analysis of the association between the expression of *DCBLD2* and EMT signal regulators, including *CDH2*, *ILK*, *MMP2*, *MMP9*, *SNAI1*, *SNAI2*, *TJP1*, *TWIST1*, *VIM*, and *ZEB2* using the GEPIA2 approach. Only targeting genes with statistically significant correlation are presented. **(D)** For the type of HNSC, COAD, CHOL, KIRP and THCA in the TCGA project, the box plot data of differential *DCBLD2* expression levels (log2 x + 1) between N stages (Normal, N1, N2, and N3) were supplied. *p < 0.05, **p < 0.01, **p < 0.001.

Next, we employed the GSCA database to elucidate the correlation between *DCBLD2* expression with tumor-related pathway activity. Results revealed that *DCBLD2* expression contributed to the activation of epithelial-mesenchymal transition (EMT) signal in 31 kinds of tumors (Figure 4A). Previous studies have demonstrated that abnormal up-regulation of EMT in cancer patients is the main factor leading to poor prognosis, metastasis, and recurrence of many types of cancer (Fan et al., 2020; Zhang et al., 2020). Correlation analysis between expression of *DCBLD2* and key regulators of the EMT signal, utilizing the TIMER 2.0 database, revealed a significant positive correlation between *DCBLD2* with *CDH1*, *TJP1*, *CDH2*, *VIM*, *SNAI1*, *SNAI2*, *TWIST1*, *MMP2*, *MMP3*, *MMP9*, *ZEB1*, *ZEB2*, *ILK,* and *RHO*, across all tumors based on the TCGA dataset (Figure 4B). Validation of these results, using the GEPIA 2.0 database, revealed that *DCBLD2* expression was positively correlated with the expression of *CDH2*, *ILK*, *MMP2*, *MMP9*, *SNAI1*, *SNAI2*, *TJP1*, *TWIST1*, *VIM,* and *ZEB2* (*p* < 0.05, *r* > 0.2) (Figure 4C). EMT is one of the key regulatory factors of cancer metastasis. Therefore, we further explored the relationship between *DCBLD2* expression with tumor M stage and N stage and found no significant effect on the tumor M stage. However, *DCBLD2* was significantly upregulated in patients with severe lymph node metastasis in CHOL, COAD, HNSC, KIRP, and THCA (Figure 4D). Collectively, these results suggested that *DCBLD2* expression is associated with activation of the EMT signal in pan-cancer and thus may promote tumor metastasis.

### DCBLD2 participates in immune regulation

**FIGURE 5:**
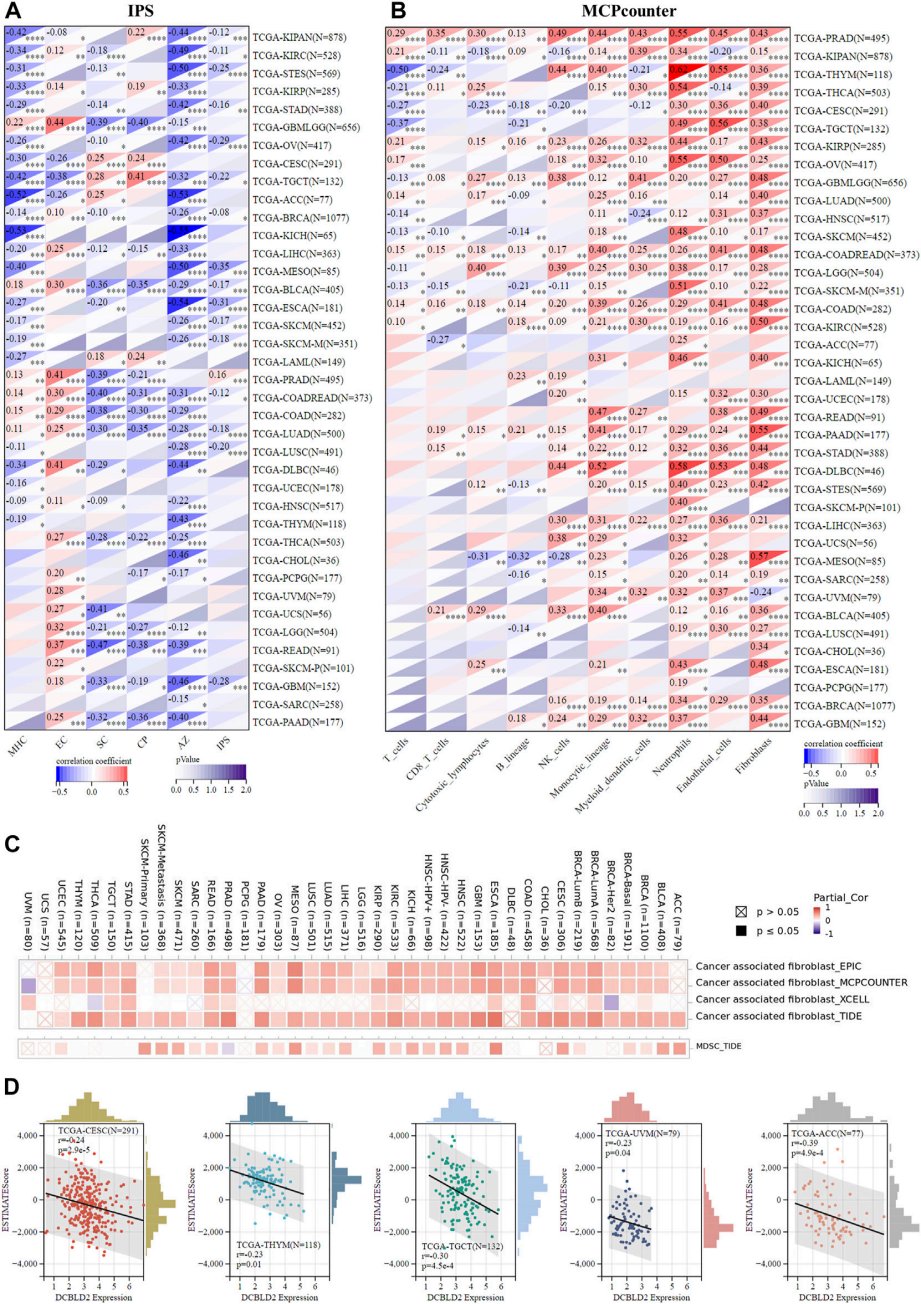
Association of *DCBLD2* with immune regulation. **(A)** Heatmap showing correlations between *DCBLD2* expression and IPS score of standardized pan-cancer data set. The MHC, EC, SC, CP, AZ, and IPS infiltration scores in each tumor were evaluated according to gene expression. **(B)** Heatmap showing correlations between *DCBLD2* expression and MCPcounter score of standardized pan-cancer data set. The T cells, CD8 T cells, Cytotoxic lymphocytes, B lineage, NK cells, Monocytic lineage, Myeloid dendritic cells, Neutrophils, Endothelial cells and Fibroblasts infiltration scores in each tumor were evaluated according to gene expression. **(C)** Heatmap showing correlations between *DCBLD2* expression and immune infiltration of cancer-associated fibroblasts (CAFs) and myeloid-derived suppressor cells (MDSCs) via TIMER 2.0 database. **(D)** Correlation analysis between *DCBLD2* expression and ESTIMATE score of standardized pan-cancer data set.

Next, we characterized the immune microenvironment of the samples according to gene expression data using multiple algorithms. Firstly, we employed IPS, which uses biomarkers of an immune response or immune tolerance for the visualization and subsequent quantification of multiple immune phenotypes (MHC, EC, SC, CP, and AZ) in tumor samples, to evaluate the immune status. This method also generates a z score that summarizes all the categories, with higher z scores in IPS indicating stronger immunogenicity in a sample. Results revealed that *DCBLD2* expression was not only inversely associated with all indexes across all tumors, except EC, but also with the final IPS score, suggesting that its upregulation may cause tumor immunosuppression (Figure 5A). Next, we used MCPcounter to quantify the relative enrichment of immune cells in heterogeneous tumor samples, and then evaluated the level of immune infiltration across 10 kinds of immune-related cells, namely T cells, CD8 T cells, natural killer cells, cytotoxic-lymphocytes, B cells, monocytic lineage, myeloid dendritic cells, neutrophils, and endothelial cells and fibroblasts. Published literature has shown that myeloid-derived suppressor cells, dendritic cells, neutrophils, and endothelial cells are key immunosuppressive cells that promote tumor progression (Liu and CAO, 2016). The correlation heat map showed a positive association between *DCBLD2* expression with infiltration of the four kinds of cells mentioned above in almost all tumors (Figure 5B). Following that, we validated the positive correlation between *DCBLD2* expression with infiltration of CAFs and MDSCs using the TIMER2.0 database (Figure 5C), then employed the ESTIMATE package in R to quantify and visualize the impact of *DCBLD2* on immune cell infiltration in specific tumor types. Scatter plots showed that levels of *DCBLD2* expression were negatively correlated with ESTIMATE scores in CESC, THYM, TGCT, UVM, and ACC (*p* < 0.05, r < -0.2) (Figure 5D).

### DCBLD2 expression is associated with therapeutic responses

**FIGURE 6:**
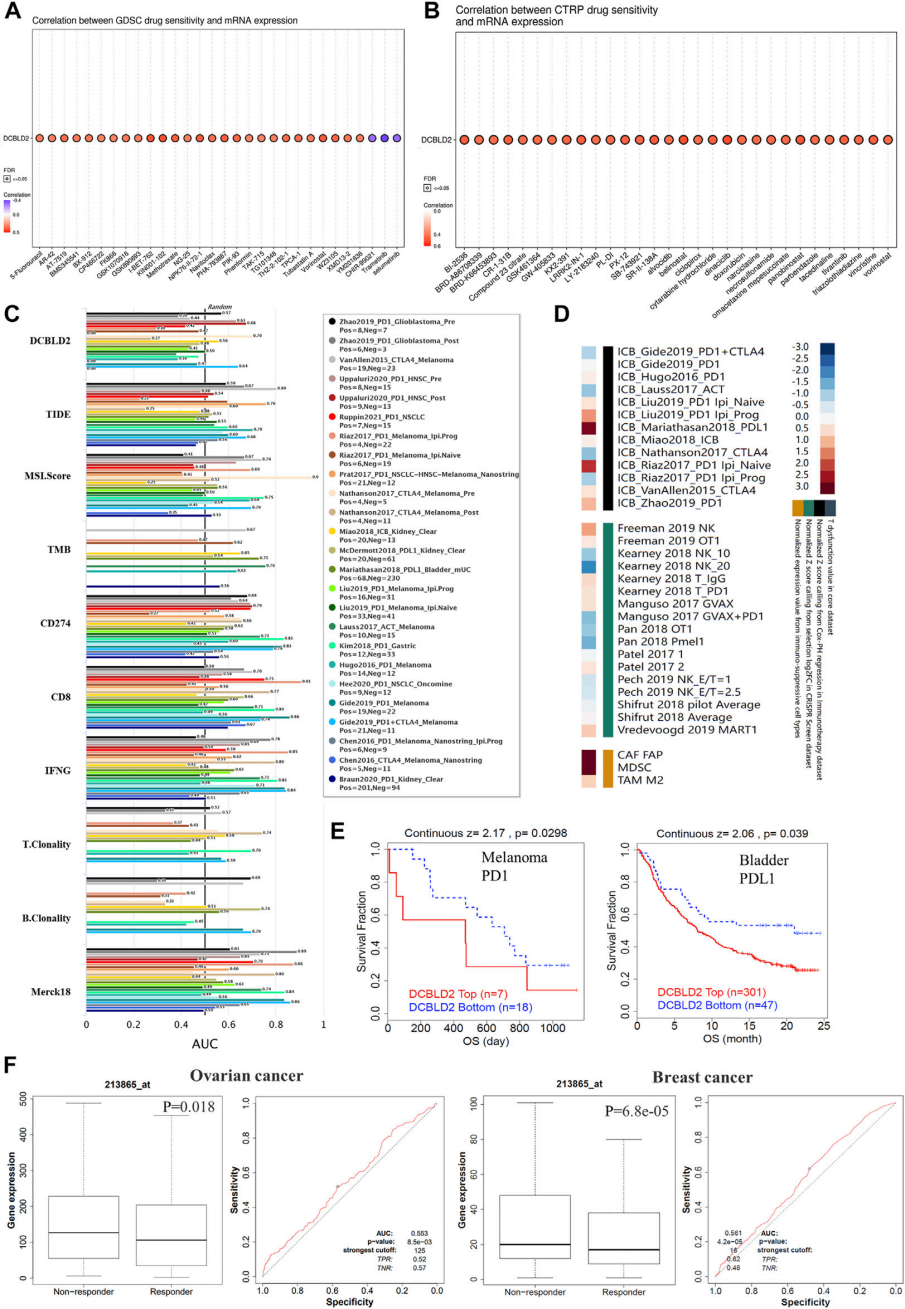
Association of *DCBLD2* expression with therapeutic responses. **(A,B)** A bubble chart showing the association between *DCBLD2* expression and IC50 value of multiple drugs in solid tumor cells, acquired from the GDSC database **(A)** and CTRP database **(B)**. The color of bubbles showing the correlation coefficient between *DCBLD2* expression level and IC50 value. The points coiled by black contours indicate that FDR < 0.05. **(C)** The bar chart showing the difference in the efficacy of DCBLD2 and standardized cancer immune evasion biomarkers in various data sets of tumor patients receiving immunotherapy. The efficacy was evaluated by the area under the receiver operating characteristic curve (AUC). **(D)** Heat maps showing the correlation between *DCBLD2* expression levels and outcome in knockout models and tumor patients receiving immunotherapy **(E)** Effect of *DCBLD2* expression on overall survival in melanoma patients treated with PD1 and bladder cancer patients treated with PDL1 (All *p* < 0.05). **(F)** Effect of *DCBLD2* expression level on chemotherapy sensitivity in breast and ovarian cancer patients and ROC curves (All *p* < 0.05).

Next, we analyzed the relationship between *DCBLD2* expression and therapeutic efficacy using a variety of solid tumors cell lines in GDSC (Figure 6A) and CTRP (Figure 6B) databases. The two databases, GDSC and CTRP, were integrated to obtain a total of about 500,000 drug effect data between 684 drugs and 1,235 cells, from which we were able to obtain the IC50 values of different types of tumor cells for various drugs. Results revealed that *DCBLD2* expression in cancer cells was positively correlated with IC50 values of many chemotherapeutic drugs, targeted drugs, and small molecular probes, including 5-Fluorouracil. Notably, high *DCBLD2* expression was associated with a weaker therapeutic effect. Next, we analyzed the potential therapeutic effects of DCBLD2 by comparing it with standardized biomarkers based upon the predicted therapeutic efficacy and patient outcome of ICB sub-cohorts. In the first, DCBLD2 alone had an AUC of the ROC > 0.5 in 7 of the 21 ICB sub-cohorts, this was accompanied by a better predictive efficacy compared with the T. Clonality and B. Clonality. Although *DCBLD2* expression was comparable to the TMB score, it was lower than MSI. Score, CD27A, TIDE, IFNG, Merck18, and CD8 (Figure 6C). Moreover, *DCBLD2* upregulation was associated with worse PD1 outcomes in glioblastoma (ICB_Zhao2019_PD1) and melanoma (ICB_Riaz2017_PD1 and ICB_Liu2019_PD1), worse CTLA4 outcomes in melanoma (ICB_Nathanson2017_CTLA4), and worse PDL1 outcomes in the bladder (ICB_Mariathasan2018_PDL1). In addition, phenotypic analysis of gene knockout mice, based on genetic screening, showed that knocking out *DCBLD2* significantly affected lymphocyte-mediated tumor killing in tumor models (Freeman 2019 NK, Vredevoogd 2019 MART1, Kearney 2018 IgG, Manguso 2017 GVAX, Kearney 2018 T_PD1 and Patel 2017 2) (Figure 6D). In addition, *DCBLD2* upregulation was related to shorter OS of melanoma and bladder cancer patients who were treated with PD1 (Figure 6E). Furthermore, we analyzed the relationship between *DCBLD2* expression level and chemotherapy sensitivity of tumor patients and found that patients with high *DCBLD2* expression in Ovarian cancer and Breast cancer were less sensitive to drug therapy (Figure 6F).

### Enrichment analysis of DCBLD2-related factors

**FIGURE 7:**
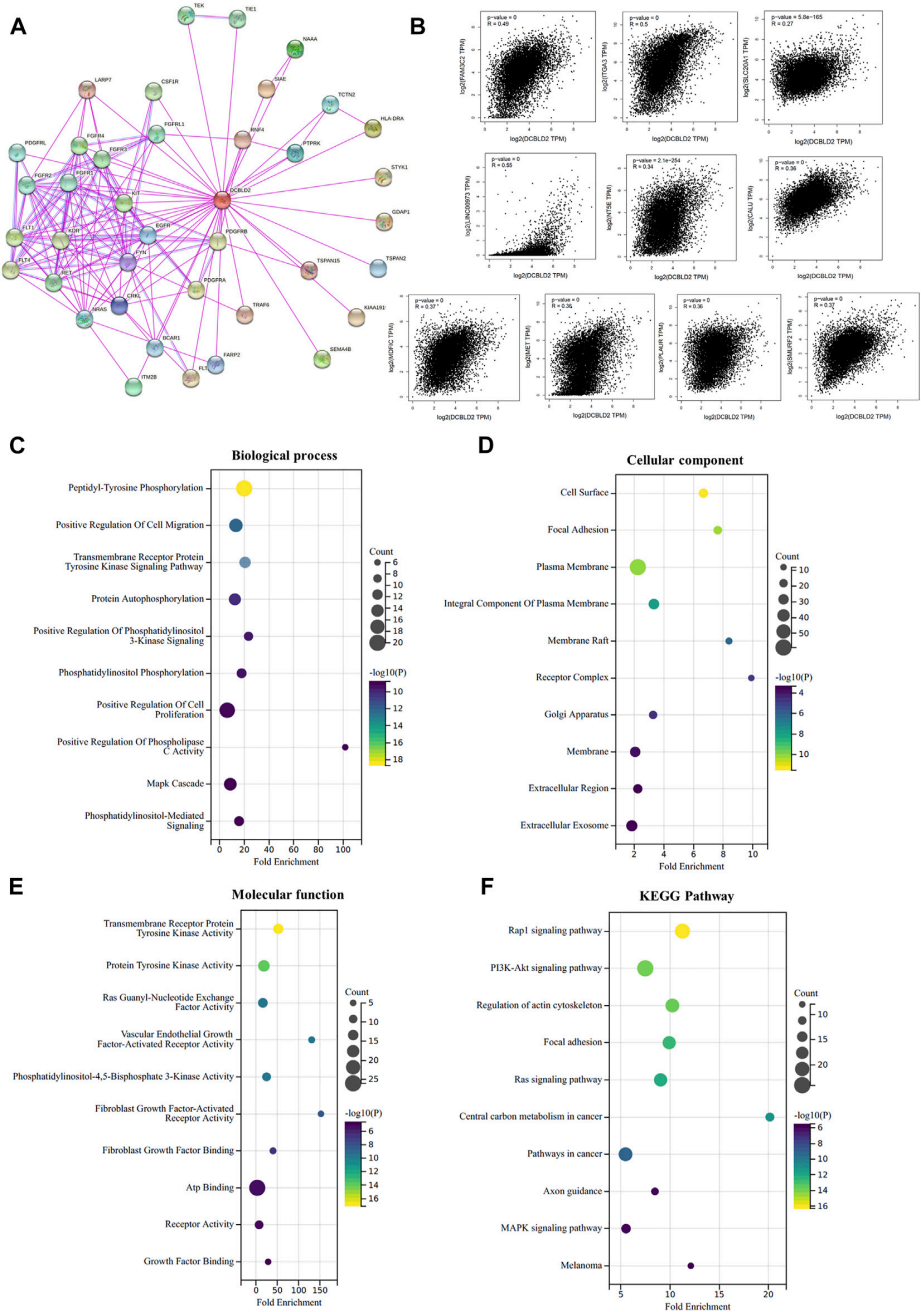
Enrichment analysis of DCBLD2-related factors. **(A)** DCBLD2-binding proteins supported by experimental results were screened using STRING database. **(B)** Scatter plot of expression correlation analysis of the top 10 *DCBLD2*-related genes, including *LINC O 0973*, *ITGA3*, *SLC20A1*, *FAM3C2*, *NT5E*, *SMURF2*, *MDFIC*, *CALU*, *MET*, and *PLAUR*. **(C–F)** GO enrichment and KEGG pathway analyses were performed based on the combine of DCBLD2-binding proteins and DCBLD2 expression-related genes. Supplymentary Figure S1: The effect of *DCBLD2* expression levels on OS, DSS or RFS in patients with colorectal, lung, breast, blood, bladder and ovarian cancers in multiple GSE datasets.

To deeper explore the molecular mechanism underlying the effects of *DCBLD2* expression level on tumor development, immune regulation and drug sensitivity, we found the potential DCBLD2 binding proteins and genes related to *DCBLD2* expression and carried out enrichment analysis. We first used the STRING tool to screen 38 possible dcBLD2-binding proteins, and the above results were supported by experimental evidence (Figure 7A). Next, we utilized the GEPIA 2.0 tool to include gene expression data of all cancer samples and selected the top 100 genes that were related to *DCBLD2* expression. The top 10 genes that were positively related to *DCBLD2* expression included *LINC O 0973*, *ITGA3*, *SLC20A1*, *FAM3C2*, *NT5E*, *SMURF2*, *MDFIC*, *CALU*, *MET* and *PLAUR* (*r* > 0.2, *p* < 0.05) (Figure 7B). We combined both datasets, and then subjected them to KEGG pathway and GO enrichment analyses. The regulation of *DCBLD2*-related genes was evaluated by biological processes, Cellular Components and molecular function. With regards to biological processes, these genes were mainly involved in peptidyl-tyrosine phosphorylation, positive regulation of cell migration, transmembrane receptor protein tyrosine kinase signalling pathway, and protein autophosphorylation, among others (Figure 7C). For cellular components, DCBLD2-related proteins were mainly located in the cell surface and plasma membrane and affected focal adhesion (Figure 7D). For molecular function, most of these genes mainly regulated the activity of various kinases and receptors, such as transmembrane receptor protein tyrosine kinase, protein tyrosine kinase, Ras guanyl-nucleotide exchange factor, vascular endothelial growth factor-activated receptor, and fibroblast growth factor-activated receptor, among others (Figure 7E). KEGG pathway data suggested that *DCBLD2* regulates the Rap1, PI3K-Akt, and Ras signalling pathways during tumor pathogenesis (Figure 7F).

## Discussion

Reported literature has revealed that DCBLD2 influences the emergence and progression of multiple diseases, including cancer (Kikuta et al., 2017; He et al., 2020; Alhamoudi et al., 2021; Coppo et al., 2021; Feng et al., 2021). Moreover, DCBLD2 has been found to interact with the receptor tyrosine kinases EGFR, VEGFR, PDGFR, and INSR (Nie et al., 2013; Feng et al., 2014; Schmoker et al., 2019; Schmoker et al., 2020). Currently, no study has investigated whether DCBLD2 regulates the immune microenvironment and pathogenesis of tumors from the pan-cancer landscape. In our study, 39 types of tumors were obtained from the TCGA data. The association between *DCBLD2* and tumor stage, prognosis, pathway activity, immune escape, and drug sensitivity was confirmed.

First, we investigated the oncogenic action of DCBLD2 in total tumor types from TCGA data. Gene differential expression analysis revealed that *DCBLD2* mRNA levels were high in nearly all TCGA cancer types, including GBM, GBMLGG, LUAD, COAD, COADREAD, STES, KIRP, HNSC, LIHC, THCA, and CHOL. Furthermore, DCBLD2 mRNA levels were linked to tumor stage in CHOL, HNSC, KIRP, LUSC, and THCA. Prognostic analysis of gene expression in 16 tumor types, including GBMLGG, LGG, LUAD, KIPAN, STAD, HNSC, GBM, KIRC, COAD, COADREAD, LIHC, MESO, PAAD, BLCA, ACC, and KICH, revealed that high *DCBLD2* expression was related to a shorter OS. We also looked at the effect of *DCBLD2* expression on DSS and DFS, and the findings showed that high expression of *DCBLD2* causes poor prognosis in a variety of tumors. The findings demonstrated that *DCBLD2* can promote the occurrence and deterioration of multiple tumors, and it has the potential to be a biomarker to predict the prognosis of patients from the perspective of pan-cancer. Moreover, there are some contradictions in this result. For example, this gene was expressed at lower levels in tumor tissues of KIRC relative to normal tissues. However, when *DCBLD2* expression was upregulated in KIRC, OS was shortened in these patients. These differences presented in individual tumors also deserve further studies to explore them in-depth.

Following the confirmation of the broad-spectrum effect of DCBLD2 on tumor prognosis, we sought to investigate the mechanism underlying this effect. In this view, we analyzed the relationship between *DCBLD2* expression and cancer-related pathway activity at the pan-cancer level, revealing that DCBLD2 activated EMT signalling in 31 different types of tumors. This result is consistent with reported clinical and preclinical studies that DCBLD2 mediates tumor metastasis in colorectal cancer and lung adenocarcinoma by stimulating EMT (He et al., 2020; Chen et al., 2021). The evaluation of the association between *DCBLD2* expression and the key regulators of EMT signalling in all tumors using TCGA data revealed significant positive correlations between *DCBLD2* expression and these genes in the majority of tumors. Moreover, mRNA levels of *DCBLD2* were correlated with the N stage. *DCBLD2* expression was higher in patients with more severe lymph node metastasis in CHOL, COAD, HNSC, KIRP, and THCA.

Previous research has shown that EMT can not only increase tumor cell migration and invasive but also initiate carcinogenic changes in the tumor microenvironment. For example, studies have reported that over-expression of EMT signal regulators (such as *TWIST1* and *MMPs*) can promote immune infiltration in TME, which promotes tumor cell immune escape (Terry et al., 2017; Singh and CHAKRABARTI, 2019) Tumor immune escape is a major contributor to tumor malignancy, poor prognosis, and treatment failure (Whiteside, 2008; Tang et al., 2016; Wu and DAI, 2017). Tumor infiltration into immune cells, on the one hand, causes T cell dysfunction, promotes tumors to evade the killing effect of the immune system, and ultimately results in tumor progression, metastasis, and chemotherapy resistance (Yu et al., 2009; Man et al., 2013). Besides, tumors can avoid immune killing via the T cell exclusion mechanism, which means that tumors prevent immune cell infiltration. T cell exclusion relies on immunosuppressive cells to play a role, including CAFs, Tregs, M2-TAMs, MDSCs, and so on (Joyce and FEARON, 2015; Komohara et al., 2016). The present investigation found that *DCBLD2* expression was inversely related to the IPS score, implying that high *DCBLD2* expression may cause tumor immunosuppression. Besides, we found a significant positive correlation between *DCBLD2* expression level and CAFs and MDSCs infiltration in almost all tumors using different algorithms. This led us to the speculation that DCBLD2 may escape immune killing via T cell exclusion, resulting in tumor malignancy and metastasis. In addition, by evaluating the effect of *DCBLD2* expression on ESTIMATE score in all tumors, we demonstrated a negative correlation between *DCBLD2* expression level with ESTIMATE score in CESC, THYM, TGCT, UVM, and ACC.

Following the validation of the influence of DCBLD2 on EMT and immune escape, its efficacy on the traditional chemotherapeutic drugs and targeted drugs was further investigated. The analysis of the relationship between *DCBLD2* expression and sensitivity to chemotherapy of all solid tumor cells showed a positive correlation of *DCBLD2* expression level in tumor cell lines with the IC50 values of many chemotherapeutic drugs, targeted drugs, and small molecular probes. Subsequently, we analyzed the role of *DCBLD2* expression levels on chemotherapy sensitivity in multiple cancer datasets. It turns out that in ovarian and breast cancer patients, high expression of the gene leads to a reduced response to drug therapy. In recent years, PD-1/PDL-1 inhibitor is the biggest breakthrough in the field of tumor therapy. This immunotherapy, which aims to reactivate the weakened immune cells of cancer patients, has achieved obvious results (Makuku et al., 2021). Our findings also revealed that high *DCBLD2* expression levels were related to poorer PD1 outcomes in glioblastoma and melanoma, poorer CTLA4 outcomes in melanoma, and poorer PDL1 outcome in the bladder. Based on the findings, we hypothesize that high *DCBLD2* expression may reduce the efficacy of chemotherapy or targeted therapy in tumor patients and that the gene may be a potential biomarker for drug efficacy evaluation or new drug development. Of course, only the correlation results of gene expression and drug sensitivity were obtained by prediction and analysis of each database, and it is essential to conduct further clinical trials or mechanistic experiments to verify the above results.

For the first time, this study: 1) Examined the effect of DCBLD2 on tumor pathological stage and prognosis from a pan-cancer perspective and discovered that DCBLD2 has a broad-spectrum activating effect on EMT signalling in all tumors. 2) Analyzed the effect of DCBLD2 on tumor immune regulation and confirmed that DCBLD2 may escape immune killing via T cell exclusion. 3) High DCBLD2 expression interferes with the efficacy of traditional chemotherapeutic drugs and emerging targeted drugs. Despite these strengths, there are some shortcomings in this study. To begin with, there is no information in these databases about *DCBLD2* gene mutation or post-translational modification. Methylation, phosphorylation, and ubiquitin, for example, may all interfere with the molecular function of DCBLD2. Secondly, we only performed bioinformatics analysis of *DCBLD2* expression, tumor stage, and prognosis in different databases, rather than *in vivo*/*in vitro* experiments. The study of the DCBLD2 mechanism at the cellular and molecular levels can clarify the role of the gene. Furthermore, this study used TCGA (RNA-seq) data, and there is a gap with protein level studies. The innovation and accuracy of this study would benefit more from the integration of proteomic level analysis in the future. Finally, while we discovered that *DCBLD2* expression was related to immune regulation in tumors, we were unable to demonstrate that DCBLD2 impacted patient prognosis and drug efficacy via immune regulation. In-depth clinical studies and molecular mechanism studies focusing on *DCBLD2* expression and immune regulation in a wide variety of tumors may help to draw a more precise conclusion.

## Conclusion

For the first time, we performed a pan-cancer analysis of DCBLD2, and the results revealed a was a statistically significant association of *DCBLD2* expression with pathological stage, prognosis, immune regulation, and chemotherapeutic and targeted drug sensitivity. Therefore, our findings indicate that DCBLD2 is an oncogenic, immunological, and prognostic factor and has the potential to be a biomarker for cancer diagnosis, drug development, and prognostic analyses.

**TABLE 1:** Online analysis tools used in this study.

Database	Website
Protter (Omasits et al. (2014))	https://wlab.ethz.ch/protter/start/
The Human Protein Atlas (Uhlén et al. (2015))	https://www.proteinatlas.org/
GeneCards (Safran et al. (2021))	https://www.genecards.org/
GPS-Prot (Fahey et al. (2011))	http://gpsprot.org/index.php
The Open Targets Platform (Carvalho-Silva et al. (2019))	https://platform.opentargets.org/
TIMER 2.0 (Li et al. (2020))	http://timer.cistrome.org/
TIDE (Fu et al. (2020))	http://tide.dfci.harvard.edu/
UALCAN (Chandrashekar et al. (2017))	http://ualcan.path.uab.edu/index.html
The ROC plotter (Fekete and Győrffy (2019))	http://www.rocplot.org
GEPIA 2.0 (Tang et al. (2019))	http://gepia2.cancer-pku.cn/
GSCA (Liu et al. (2018a))	http://bioinfo.life.hust.edu.cn/GSCA/
cBioPortal (Gao et al. (2013))	http://www.cbioportal.org/
STRING (Szklarczyk et al. (2021))	https://string-db.org/
DAVID (Huang et al. (2009))	https://david.ncifcrf.gov/

## Data Availability

The datasets presented in this study can be found in online repositories. The names of the repository/repositories and accession number(s) can be found in the article/Supplementary Material.

## Abbreviations

TME, Tumor microenvironment; MDSCs, Myeloid-derived suppressor cells; DCs, Dendritic cells; CAFs, Cancer-associated fibroblasts; DCBLD2, Discoidin, CUB And LCCL Domain Containing 2; OS, Overall survival; DSS, Disease Specific Survival; EMT, Epithelial-Mesenchymal Transition; TCGA, The Cancer Genome Atlas; GEO, Gene Expression Omnibus; CRC, Colorectal cancer; ECM, Extracellular matrix; CAFs, Cancer-associated fibroblasts.
