# Supplementary figures and images for: Pan-cancer analyses identify DCBLD2 as an oncogenic, immunological, and prognostic biomarker
Source: Front Pharmacol. 2022 Aug 11;13:950831. doi: 10.3389/fphar.2022.950831 (PMC9403722; doi:10.3389/fphar.2022.950831)

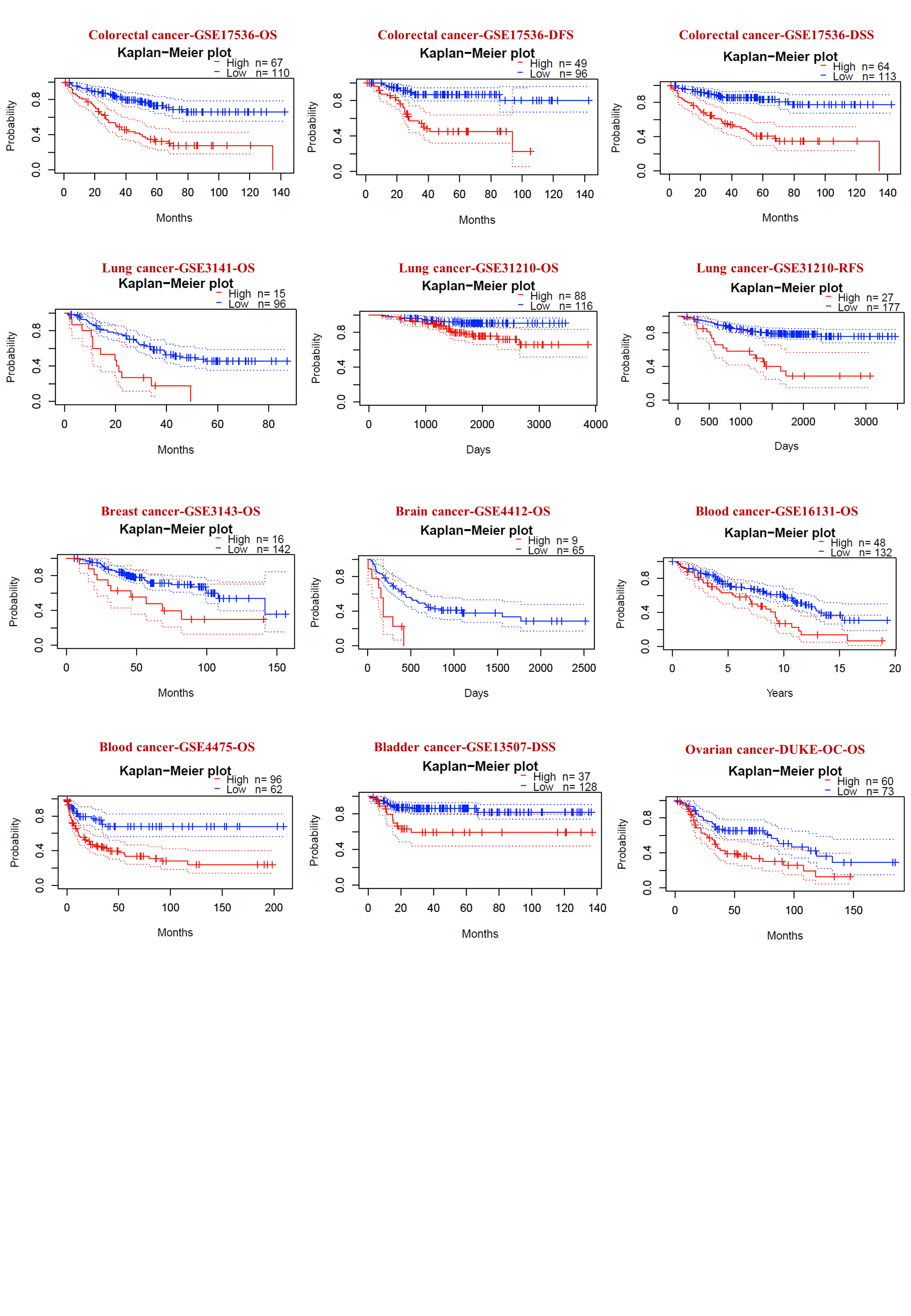

Supplement: Supplementary file 1 [file Image1.TIF]
